# Simultaneous Exposure of Different Nanoparticles Influences Cell Uptake

**DOI:** 10.3390/pharmaceutics14010136

**Published:** 2022-01-06

**Authors:** Isa de Boer, Ceri J. Richards, Christoffer Åberg

**Affiliations:** Groningen Research Institute of Pharmacy, University of Groningen, Antonius Deusinglaan 1, 9713 AV Groningen, The Netherlands; i.de.boer.11@student.rug.nl (I.d.B.); c.j.richards@rug.nl (C.J.R.)

**Keywords:** nanomedicine, endocytosis, synergy, antagonism, flow cytometry

## Abstract

Drug delivery using nano-sized carriers holds tremendous potential for curing a range of diseases. The internalisation of nanoparticles by cells, however, remains poorly understood, restricting the possibility for optimising entrance into target cells, avoiding off-target cells and evading clearance. The majority of nanoparticle cell uptake studies have been performed in the presence of only the particle of interest; here, we instead report measurements of uptake when the cells are exposed to two different types of nanoparticles at the same time. We used carboxylated polystyrene nanoparticles of two different sizes as a model system and exposed them to HeLa cells in the presence of a biomolecular corona. Using flow cytometry, we quantify the uptake at both average and individual cell level. Consistent with previous literature, we show that uptake of the larger particles is impeded in the presence of competing smaller particles and, conversely, that uptake of the smaller particles is promoted by competing larger particles. While the mechanism(s) underlying these observations remain(s) undetermined, we are partly able to restrain the likely possibilities. In the future, these effects could conceivably be used to enhance uptake of nano-sized particles used for drug delivery, by administering two different types of particles at the same time.

## 1. Introduction

Using nano-sized carriers to deliver drugs holds great promise to cure a range of diseases [1,2,3,4], including cancers [5,6,7,8,9,10,11]. In this endeavour, it is pertinent to understand how many carriers enter cells because the intracellular dose typically determines the therapeutic benefit [12,13,14]. Equally important is the fact that the intracellular dose determines (potential) adverse effects when the carrier enters healthy cells (off-target effects), as well as how long the object remains in the body (clearance).

Nanoparticle internalisation by cells has been extensively studied, with particular attention paid to the internalisation pathways utilised by particles [12,13,14,15,16,17,18] and how they depend upon particle properties such as size [12,13,16,17,18,19,20], shape [12,13,16,17,18,20] and biomolecular corona [12,18]. These studies have been conducted in a range of different cell systems: simple adherent cells such as HeLa [21,22,23,24], U-2 OS [25] and A549 [26,27,28]; red blood cells [29,30]; barrier-forming cells such as Caco-2 [31], human umbilical vein endothelial cells [32] and bEnd3 [33]; three-dimensional cell systems [34,35]; and ex vivo tissue [36], to name a few.

An aspect that is less well-explored, however, is the effect on uptake of one particle in the presence of another, when both particles are exposed to cells at the same time. In this context, an increase in uptake of both gold and iron oxide particles has been described when the two particles are exposed together to J774A.1 murine macrophages [37]. Similarly, it has been reported that uptake of a number of different nanoparticles increases when the nanoparticles are exposed to cells together with functionalised nanoparticles [38,39]. An increased uptake has also been described in vivo when nanoparticles are simultaneously administered with nanoparticles self-assembled from amphiphilic peptides [40]. Furthermore, a similar effect has been noted for uptake of micron-sized particles, where polystyrene particles increase the uptake of poly(lactic-*co*-glycolic) acid (PLGA) particles by NR8383 rat alveolar macrophage cells at higher concentrations [41]. In contrast, it has been reported that there is no difference in uptake of nano-sized (around 60 nm) silica by J774A.1 mouse macrophages when cells are simultaneously exposed to micron-sized (around 900 nm) particles of the same material [42]. For completeness, we note that the effect of non-particle species on nanoparticle uptake has also been described; we refer to a recent review for more information [43].

Other studies have described changes to cellular function when exposing two different nanoparticles to cells simultaneously, something which could be due to changes in uptake, but which could also be due to other effects. For example, it has been reported that simultaneous exposure of carbon black and iron oxide (Fe_2_O_3_) particles to human epithelial A549 cells induces protein and lipid oxidation, although no such effect is observed when the cells are exposed to the particles individually [44]. The induction of oxidation was, however, not ascribed to a difference in uptake [44]. Similarly, it has been reported that silica and titania nanoparticles activate mouse bone marrow-derived macrophages when exposed together, but not when exposed alone (at the same concentration), although a large part of the effect could be due to a change in colloidal stability when the two particles are dispersed together [45]. Furthermore, the effect of simultaneous exposure of iron oxide (Fe_2_O_3_) and polymorphous silica particles on cell viability, reactive oxygen species production, mitochondrial membrane potential and intracellular glutathione content in human epithelial A549 cells has also been investigated [46]. On a final note, we mention that when considering effects due to multiple particles, one should keep in mind the possibility of a non-linear relationship between response and dose, as recently discussed in the broader context of synergy [47].

All studies that we are aware of report an increase in uptake when two nanoparticles are exposed to cells at the same time. An exception is the work of Li et al., who investigated the uptake of silica nanoparticles of different sizes (50, 80, 100 and 150 nm) by HeLa cells [48]. Similar to previous studies, they observed an increase in uptake of smaller particles in the presence of larger particles but, interestingly, also a decrease in uptake of larger particles in the presence of smaller. It is worth noting that these experiments were performed in serum-free medium, which implies the absence of a biomolecular corona on the particles [49]. The lack of a corona on silica particles specifically may lead to cell membrane damage and other cellular effects [50], although there are no clear signs of this in the report by Li et al. [48].

Here, we were interested in testing whether the observation that the uptake of smaller particles is increased in the presence of larger particles while the uptake of larger particles is decreased in the presence of smaller particles remains true also for particles covered by a biomolecular corona. We exposed cells simultaneously to two different sizes of carboxylated polystyrene nanoparticles labelled with two different fluorescent dyes such that we can follow the uptake of both particles concurrently. The particles were exposed to cells in medium with serum, implying the formation of a biomolecular corona on the particles. Like Li et al. [48], we observe that the smaller particles impede the uptake of the larger, while the larger particles promote the uptake of the smaller but now in the presence of a biomolecular corona. We also investigate the uptake at a single-cell level.

## 2. Materials and Methods

### 2.1. Materials

40 nm yellow/green and 100 nm red fluorescently labelled carboxylated polystyrene nanoparticles were purchased from ThermoFisher (Eugene, OR, USA) and were used without further chemical modification. HeLa cells were acquired from American Type Culture Collection (ATTC; Manassas, VA, USA; CCL-2TM, lot no. 61647128). Dulbecco’s Modified Eagle Medium (DMEM; Gibco, Life Technologies, Eugene, OR, USA), Dulbecco’s Phosphate Buffered Saline (PBS; Gibco, Life Technologies, Eugene, OR, USA), foetal bovine serum (FBS; Gibco, Life Technologies, Eugene, OR, USA) and Trypsin-EDTA (Gibco, Life Technologies, Eugene, OR, USA) were purchased from ThermoFisher (Eugene, OR, USA).

### 2.2. Cell Culture

HeLa cells were cultured in DMEM supplemented with 10% FBS (cDMEM) at 37 °C under a humidified atmosphere and 5% CO_2_. Mycoplasma tests were performed regularly and showed no contamination.

### 2.3. Flow Cytometry

#### 2.3.1. Nanoparticle Dispersion

Nanoparticle dispersions were freshly prepared under sterile conditions and the stocks were vortexed before usage. When studying the uptake of the 100 nm particles in the presence of competing 40 nm particles, an initial dispersion of the 100 nm particles was prepared by diluting the 100 nm stock in cDMEM and vortexing. Subsequently, this dispersion was divided up and different amounts of 40 nm dispersion added to the different samples (depending upon the desired 40 nm concentration, either the stock dispersion was directly added or a dispersion of the 40 nm particles was first prepared) and the final dispersions vortexed. When studying the uptake of the 40 nm particles in the presence of competing 100 nm particles, the roles of the two particles were reversed.

#### 2.3.2. Nanoparticle Exposure

Cells were seeded in 24-well plates (around 100,000 cells/well) and left to adhere to the substrate by incubation for a day at 37 °C under a humidified atmosphere and 5% CO_2_ before nanoparticle exposure. The cells were exposed to the particles by removing the medium from the cells and replacing it with the nanoparticle dispersions, after which the cells were further incubated for a day at 37 °C under a humidified atmosphere and 5% CO_2_.

#### 2.3.3. Flow Cytometry

The (nanoparticle-containing) medium was removed and the cells washed once with cDMEM and twice with PBS, after which the cells were detached by addition of trypsin and incubation for 5 min at 37 °C under a humidified atmosphere and 5% CO_2_. The cells were subsequently harvested, spun down for 5 min at 300 RCF and resuspended in PBS for measurement.

Cells were measured using a NovoCyte Quanteon flow cytometer. The 40 nm yellow/green particles were excited at 488 nm and the emission was collected at 530/30 nm, while the 100 nm red particles were excited at 561 nm and the emission was collected at 615/20 nm. Around 15,000 cells were measured per sample.

Cells were differentiated from debris in terms of forward scattering and side scattering and single cells further differentiated from (potential) cell agglomerates using forward scattering area and height. The arithmetic mean was used when reporting fluorescence averaged over cells.

### 2.4. Microscopy

The cells were seeded on 35 mm petri dishes with glass bottom microwells (no. 1.5 coverslip, MatTek, Ashland, MA, USA) 2 days prior to imaging. Nanoparticle dispersions were prepared by adding both the 40 and 100 nm particles to cDMEM to obtain concentrations of 100 and 20 µg/mL or 6.25 and 80 µg/mL for the 40 and 100 nm particles, respectively. The medium in the petri dishes was removed and replaced with nanoparticle dispersion 24 h before imaging. Immediately prior to imaging, the particle dispersion was removed from the dishes and replaced with Live Cell Imaging Solution (Invitrogen, Waltham, MA, USA). The dishes were placed on a CellDiscoverer 7 (Zeiss, Oberkochen, Germany) microscope with 37 °C heating and 5% CO_2_. Images at a lower magnification were obtained using a 50× plan apochromatic water immersion objective (used with autocorrection rings) in phase gradient contrast and epifluorescence mode in combination with an Axiocam 506 camera (Zeiss, Oberkochen, Germany). A 470 nm LED with the AF488 filter setting was used to image the 40 nm yellow/green particles, while the 100 nm red particles were imaged using a 590 nm LED and the AF568 filter. High resolution images were taken in confocal mode with the LSM900 and AiryScan 2 detector at 50× magnification with the same filter settings, but instead exciting the two particles with a 488 and 566 nm laser, respectively.

### 2.5. Statistical Analysis

Statistical differences between the fluorescence of cells exposed to different particle concentrations were assessed using the nonparametric rank-based Mack–Skillings test with an equal number of observations [51], with independent experiments as blocking factor and replicate samples as multiple observations. All experiments (including those reported in the Supplementary Material) were used in the test. The significance level was set to 5%.

## 3. Results and Discussion

To study the effect of one particle on the cellular uptake of another, we used a model system consisting of two different sizes of carboxylated polystyrene particles exposed to HeLa (adenocarcinomic human cervical epithelial) cells. We chose these particles because we have ample previous data on their uptake by cells to support our arguments [26,27,28,52,53,54,55,56] and because they disperse well in biological media [26,27] and do not exhibit any known adverse effects on cells [27,57,58,59]. Specifically, we used 40 nm diameter particles labelled in yellow/green and 100 nm particles labelled in red (the sizes correspond to the nominal diameters reported by the manufacturer). The particles were dispersed in cell medium supplemented with serum and the dispersions characterized by dynamic light scattering (Table S1 and Figure S1). The results show that both sizes remain well dispersed when dispersed separately, consistent with previous literature [26,27], and that mixing the two particles in medium with serum does not cause agglomeration. The use of serum in the medium implies the formation of a biomolecular corona [49] on the particles [60]. In this context, we note that the amount of serum is in excess compared to the (total) nanoparticle surface area (Table S2). Throughout, we report particle concentrations in particle mass per volume of dispersion because this is how the dispersions were prepared; additionally, molar concentrations, estimated using the nominal diameters, are reported parenthetically.

To quantify the number of particles taken up by cells we used flow cytometry, which allows us to measure the fluorescence corresponding to both particles on an individual cell basis for thousands of cells [61]. We exposed the particles to the cells for 24 h and measured around 15,000 cells, which is enough to quantify the average cell fluorescence to within less than 1% [27,61]. There are no reported cell export processes for these particles in simple cell lines [27,62]; thus, cell fluorescence levels are representative of uptake as opposed to a combination of uptake and export. The results show inter-experimental variability in terms of absolute numbers, while the trends are reproducible. Rather than averaging over repeat experiments we therefore choose to present representative experiments in the main text, with repeat experiments relegated to the Supplementary Material. Regardless, all experiments were included in the statistical analysis. Our measurements include both the intracellular particles, as well as those strongly adsorbed to the outer cell membrane (the cells undergo centrifugation before measurement; thus, *loosely* adsorbed particles are unlikely). However, already within 3 h, the measured value is dominated by intracellular particles [28,61]; thus, we deem that the contribution from adsorbed particles is negligible after the 24 h exposure we employed here. Another point bearing in mind is that flow cytometry measures cell fluorescence intensity, something which is obviously related to the number of particles, but in practice has to be interpreted as giving the number of particles in “arbitrary units”. In particular, the signal corresponding to the two particles cannot be compared in absolute terms, given their different fluorescence loading and spectra. This has no bearing on our conclusions, which are all based on relative comparisons (trends).

**Figure 1:**
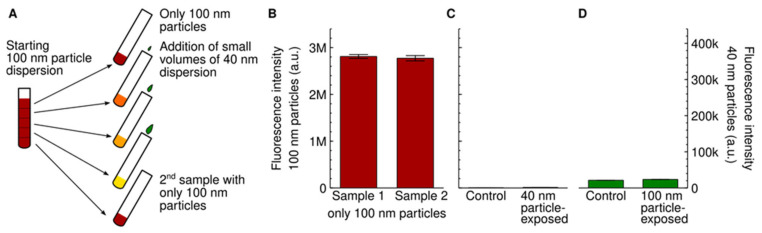
Experimental setup. (**A**) Schematic showing the dispersion procedure we used to ensure that the concentration of the 100 nm particles was the same for all samples. A first dispersion of the 100 nm particles was prepared and then divided up. To the resulting samples, different small volumes of a 40 nm particle dispersion were added. The first and last sample were left without 40 nm particles to serve as controls. Note that the colours are schematic only. For the experiments where we investigated the effect of competing 100 nm particles on the uptake of the 40 nm particles (Figure 4 and Figures S4 and S5), the role of the two particles should be reversed. See Methods for further details. (**B**) Nanoparticle fluorescence of cells exposed to the two different control dispersions of the 100 nm particles mentioned in panel A (first and last sample; 20 μg/mL or 0.060 nM). The similarity of the signal is consistent with the two control samples having the same nanoparticle concentration. (**C**,**D**) Lack of cross-talk between the two particle signals. (**C**) Cells were exposed to only the 40 nm particles (100 μg/mL; 4.7 nM) and the fluorescence intensity measured at the same wavelengths where we measured the 100 nm particles (left axis). There is a low background signal (right bar), but this is comparable to control cells not exposed to any particles at all (left bar). Note that the scale has been set to be relevant for the later results (Figure 2A and Figures S2 and S3). (**D**) Cells were exposed to only the 100 nm particles (80 μg/mL; 0.24 nM) and the fluorescence intensity measured at the same wavelengths where we measure the 40 nm particles (right axis). There is a low background signal (right bar), but this is comparable to control cells not exposed to any particles at all (left bar). Note that the scale has been set to be relevant for the later results (Figure 4A and Figure S4). Overall, the results show that the contribution of one of the nanoparticles to the fluorescence when the other nanoparticle is measured is similar to control cells. For panels (**B**–**D**), results are presented as the mean ± its standard error over three samples.

We started by studying whether the uptake of the 100 nm particles would be affected by the presence of competing 40 nm particles. It is then of utmost importance that the concentration of the 100 nm particles is kept constant, because otherwise it is impossible to tell whether any potential difference in the uptake of the 100 nm particles is due to the competing 40 nm particles, or simply due to a (slightly) variable 100 nm particle concentration. We tried a few different variations to ensure a constant concentration and finally settled on the following procedure: We used a single dispersion of the 100 nm particles to prepare all samples, diluting different (small) amounts of a 40 nm particle dispersion into them. Figure 1A illustrates this procedure. In principle, the addition of different volumes of the 40 nm particle dispersion implies a slightly different dilution of the original 100 nm particle dispersion. However, the largest volume we added was 8 µL to 2 mL of the original 100 particle dispersion, which amounts to only 0.4%. This minute dilution is completely negligible compared to the effects we will show below.

As an extra precaution, we additionally prepared two dispersions with only the 100 nm particles (at a concentration of 20 μg/mL; 0.060 nM). One of these was the first one to be prepared from the starting 100 nm particle dispersion, while the second was the last one (Figure 1A). Naturally, if the original dispersion was well-mixed, then these dispersions would be identical. After exposing cells to these dispersions, we subsequently assessed whether the cells indeed exhibited the same fluorescence. Figure 1B shows that the average cell fluorescence is similar for the two (nominally the same) dispersions, consistent with the dispersion procedure resulting in well-mixed samples. We repeated this test for all experiments, and for all results reported here the two dispersions gave a similar fluorescence when exposed to cells (see insets in figures below).

Since we wanted to simultaneously assess the cellular uptake of two particles with different fluorescent properties, it is imperative that the signal due to one is not present when quantifying the other and vice versa (no cross-talk). Thus, we exposed cells to only one of the particles, at the highest concentration used for the rest of the study, and verified that the fluorescence intensity corresponding to the other particle was negligible compared to the effects we measure (Figure 1C,D).

Having set up the methodology, we next performed an initial experiment where we measured the uptake of the 100 nm particles (20 μg/mL; 0.060 nM) in the presence of varying concentrations (3–12 μg/mL; 0.15–0.59 nM) of the competing 40 nm particles. Here and below, we will present our results as the average cell fluorescence of the 100 nm particles as the concentration of the competing 40 nm particles is varied. We use two different scales for the concentration: the 40 nm particle mass concentration as well as the particle number ratio (number of 40 to number of 100 nm particles). Under these conditions, there is no substantial effect on the uptake of the 100 nm particles when the concentration of the competing 40 nm particles is varied (Figure S2). This is in contrast to previous literature, where a decrease in uptake of larger particles in the presence of competing smaller particles was observed for number concentration ratios between 0–8 [48], similar to here.

**Figure 2:**
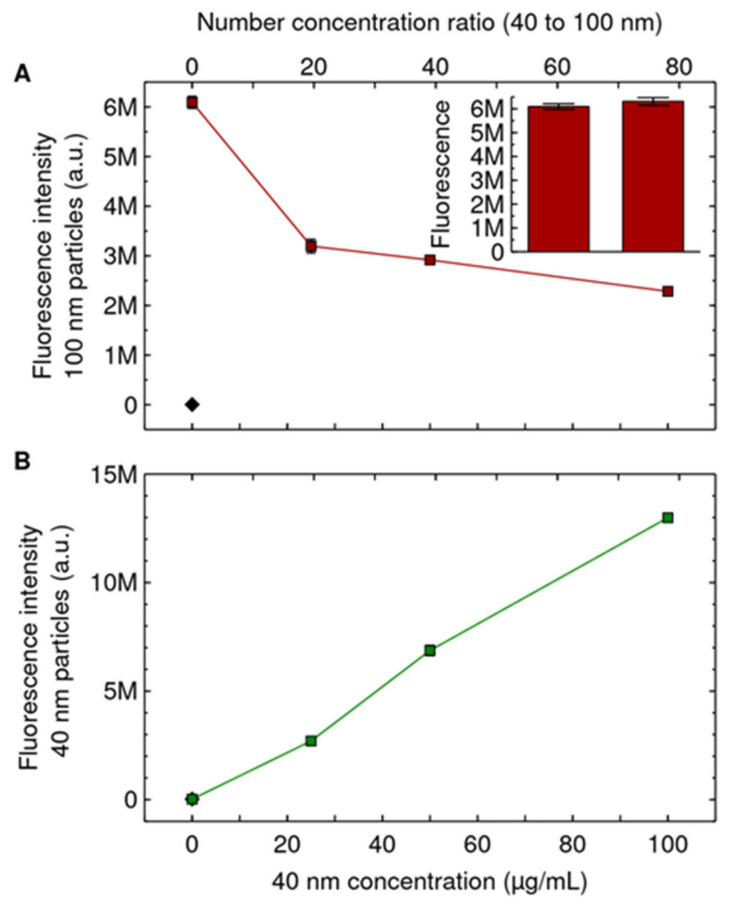
Competing 40 nm particles impede uptake of the 100 nm particles. Cells were exposed for 24 h to both 100 and 40 nm particles simultaneously. The 100 nm particle concentration was kept constant at 20 μg/mL (0.060 nM), while the concentration of the 40 nm particles was varied (horizontal axes). (**A**) Uptake of the 100 nm particles, showing that as the concentration of the competing 40 nm particles is increased, the uptake of the 100 nm particles decreases. (Inset) Cells exposed to the two dispersions of only the 100 nm particles (20 μg/mL; 0.060 nM) as a control for having achieved a similar concentration of the 100 nm particles (*cf.* Figure 1A,B). Same *y* axis as the main figure. (**B**) Uptake of the 40 nm particles, showing that the uptake of the competing 40 nm particles increases as their concentration is increased. Results are presented as the mean ± its standard error over 3 samples (most error bars are, however, smaller than the data symbols and are hence not visible). Diamonds corresponds to control cells (not exposed to either of the particles). Repeat experiments are shown in Figure S3. A Mack–Skillings test with independent experiments as blocking factor and replicate samples as multiple observations shows a statistically significant difference with competing 40 nm particle concentration.

We next wanted to determine whether the uptake rate was also unaffected for higher concentrations of the competing 40 nm particles. We thus exposed cells to the same concentration of the 100 nm particles (20 μg/mL; 0.060 nM) but used higher concentrations of the competing 40 nm particles (25–100 μg/mL instead of 3–12 μg/mL; 1.2–4.7 nM instead of 0.15–0.59 nM). In contrast to what was observed at lower concentrations (Figure S2), the uptake of the 100 nm particles was substantially decreased for higher concentrations of the competing 40 nm particles (Figure 2A), being lowered by up to 60% at the highest concentration. We note that even if there was a substantial contribution of the 40 nm particles to the signal corresponding to the 100 nm particles (which Figure 1C shows that there is not), that would amount to an *increase* of the signal with increasing concentration of the competing 40 nm particles; consequently, this could not explain the *decrease* we observe in Figure 2A. Furthermore, as already noted, the slight difference in 100 nm particle concentration implied by our dispersion procedure (Figure 1A) is completely negligible (at most 0.4%) compared to the 60% decrease observed in Figure 2A.

We also note that while the uptake of the 100 nm particles was impeded, we did not observe any obvious simultaneous impediment to the uptake of the competing 40 nm particles (Figure 2B). We interpret this to mean that there is no saturation of cell membrane “adsorption sites” or cell “internalisation portals” [28,63]; if these were saturated, then we would expect the uptake of the competing 40 nm particles to also be impeded (“self-competition”) contrary to observation (Figure 2B). This makes direct competition between the two particles less likely as a mechanism for the impediment of 100 nm particle uptake by the competing 40 nm particles.

**Figure 3:**
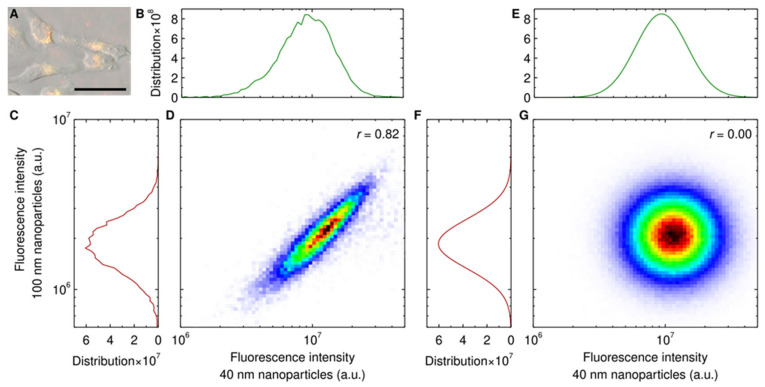
Uptake of the 40 and 100 nm particles at individual cell level. (**A**–**D**) Experimental results. Cells were exposed for 24 h to both the 100 nm (20 μg/mL; 0.060 nM) and 40 nm (100 μg/mL; 4.7 nM) particles simultaneously (conditions correspond to the highest 40 nm particle concentration in Figure 2 and S3). (**A**) Microscopy of cells. (Grey) Phase-gradient contrast microscopy image showing the contour of the cells. (Red) 100 nm and (green) 40 nm particles. (Yellow) Overlap of the two particles. Note that the fluorescence intensity of the two particles is different; thus, the results cannot be interpreted in absolute terms. Scale bar corresponds to 50 μm. The results show that cells take up both nanoparticles. (**B**–**D**) Fluorescence of cells measured using flow cytometry. Distribution of cell fluorescence corresponding to the (**B**) 40 nm and (**C**) 100 nm particles. (**D**) Two-dimensional distribution of the two fluorescences. The results show a strong correlation between a cell having taken up one of the nanoparticles with it having taken up the other nanoparticle. (**E**–**G**) Theoretical distributions derived from fits to the experimental data (panels (**B**–**D**)). (**E**,**F**)**,** Log normal distribution fits to the distribution of individual fluorescences (panels (**B**,**C**)). (**G**) Two-dimensional distribution derived under the assumption that the two fluorescences are independent. To evaluate the distribution, 10^6^ random samples were drawn from each of the two individual distributions (panels (**E**,**F**)). The simulated results do not show any correlation between a cell having taken up one of the nanoparticles and it having taken up the other nanoparticle, in stark contrast to the experimental results (panel (**D**)). We can therefore conclude that experimentally the uptake of one particle is not statistically independent of the uptake of the other. Panels use the same scale where possible. All distributions have been normalised such that their integral is 1. *r* denotes Pearson’s correlation coefficient, which was evaluated in the interval shown to not bias the result from outliers.

Results such as those shown in Figure 2and Figure S2 are cell population averages and thus do not provide information on whether the impediment of uptake occurs at an individual cell level. In other words, does each cell (on average) take up fewer of the 100 nm particles as the concentration of the competing 40 nm particles increases, or do particular cells take up fewer of the 100 nm particles? To answer this question, we also examined the (two-dimensional) distribution of the fluorescence stemming from the two particles (Figure 3D). We do not observe any subpopulations, but rather it appears that all cells take up both particles, to varying degrees. Fluorescence imaging under the same conditions likewise shows that cells contain both types of particles (Figure 3A), thus qualitatively confirming the flow cytometry results. Overall, it thus seems likely that the impediment of 100 nm particle uptake by the competing 40 nm particles occurs for all cells of the population.

To make this discussion more precise, we considered whether the uptake of the 100 nm particles and the competing 40 nm particles are independent in the statistical sense. In this case, statistical independence is equivalent to the two-dimensional distribution function of the two fluorescence intensities being the product of the distribution functions of the two fluorescences individually [64]. We thus determined the distribution functions of the two fluorescence intensities individually (Figure 3B,C). Both of these distribution functions are well described by a log normal distribution (Figure 3E,F) [27,62,65], which allowed us to approximate their product as the product of two log normal distributions (Figure 3G). The (approximate) product of the distribution functions of the two fluorescences individually (Figure 3G) is markedly different from the two-dimensional distribution function of the two fluorescence intensities measured experimentally (Figure 3D). This shows that the two fluorescence intensities are not statistically independent.

As a further confirmation, we also evaluated the (Pearson) correlation coefficient. If the two fluorescence intensities were independent, then the correlation coefficient would be 0 (indeed, the product of two gamma distributions shown in Figure 3G exhibits a vanishing correlation coefficient) [64]. The experimental data, however, has a correlation coefficient of 0.82 (Figure 3D) thus confirming that the two fluorescence intensities are not independent.

Having shown an impediment to the uptake of 100 nm particles in the presence of competing 40 nm particles, we next reversed the situation, that is, we considered the uptake of the 40 nm particles in the presence of competing 100 nm particles. Since in this case it was most important to keep the concentration of the 40 nm particles constant, we started with a single dispersion of the 40 nm particles (the reverse of Figure 1A). We used a fixed concentration of the 40 nm particles (6.25 μg/mL; 0.30 nM) and varied the concentration of the competing 100 nm particles (20–80 μg/mL; 0.060–0.24 nM).

**Figure 4:**
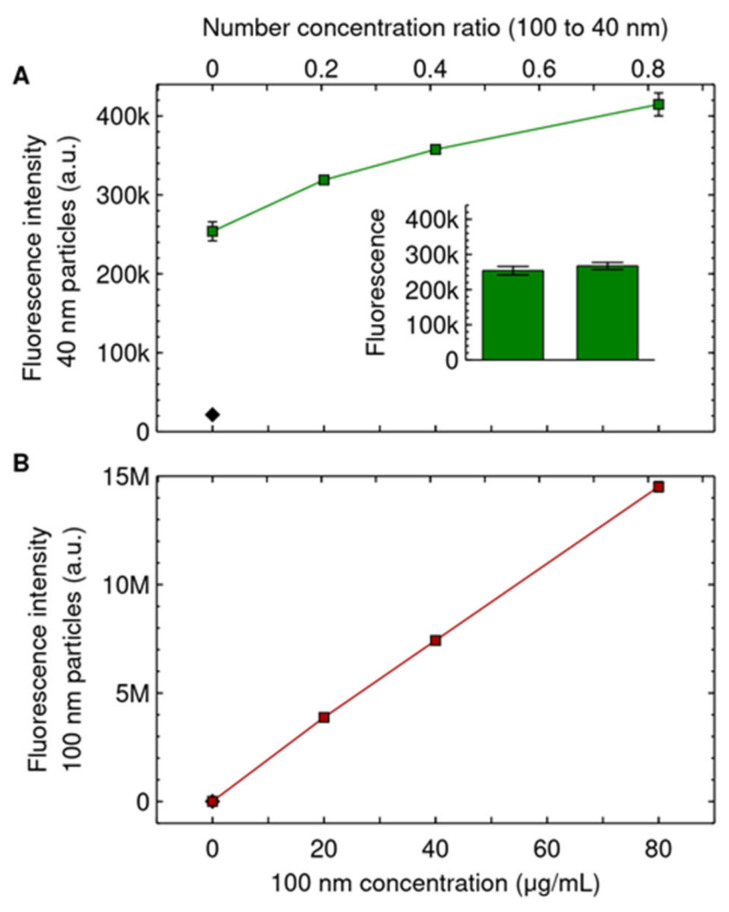
Competing 100 nm particles promote uptake of the 40 nm particles. Cells were exposed for 24 h to both 40 and 100 nm particles simultaneously. The 40 nm particle concentration was kept constant at 6.25 μg/mL (0.30 nM), while the concentration of the 100 nm particles was varied (horizontal axes). (**A**) Uptake of the 40 nm particles, showing that as the concentration of the competing 100 nm particles is increased, the uptake of the 40 nm particles increases. (Inset) Cells exposed to the two dispersions of only the 40 nm particles (6.25 μg/mL; 0.30 nM) as a control for having achieved a similar concentration of the 40 nm particles (*cf.* Figure 1A–B, but with the role of the 40 and 100 nm particles reversed). Same *y* axis as the main figure. (**B**) Uptake of the 100 nm particles, showing that the uptake of the competing 100 nm particles increases as their concentration is increased. Results are presented as the mean ± its standard error over 3 samples (most error bars are, however, smaller than the data symbols and are hence not visible). Diamonds corresponds to control cells (not exposed to either of the particles). Repeat experiments are shown in Figure S4. A Mack–Skillings test with independent experiments as blocking factor and replicate samples as multiple observations shows a statistically significant difference with competing 100 nm particle concentration.

Figure 4A shows that as the concentration of the competing 100 nm particles is increased, the uptake of the 40 nm particles increases by up to 60% at the highest concentration. Such an increase could have been due to the 100 nm particles contributing to the signal corresponding to the 40 nm particles, but this contribution is negligible (Figure 1D). Furthermore, as already noted, the slight difference in 40 nm particle concentration implied by our dispersion procedure (Figure 1A) is completely negligible (at most 0.4%) and, in any case, would have led to a *decrease* at higher concentrations, contrary to observations. Our observations are similar to previous literature, which also reported an increase in the uptake of smaller particles in the presence of larger particles, at similar number concentration ratios [48].

We also observe that the uptake of the competing 100 nm particles remained linear with respect to concentration to a good approximation (Figure 4B). Thus, as in the opposite situation (Figure 2B), there is no saturation of adsorption/internalisation. Furthermore, this observation makes certain potential mechanisms for the promotion of uptake less probable. For example, if the competing 100 nm particles promoted general endocytosis (or a particular endocytic mechanism) then we would expect that the uptake of the competing 100 nm particles themselves would also increase at higher concentrations and become non-linear (“self-induction”). However, this is not what is observed (Figure 4B).

As before, we also studied the effect at the level of individual cells, confirming that all cells take up both particles, to varying degrees, and that the uptake of the two particles is not statistically independent (Figure S5).

**Figure 5:**
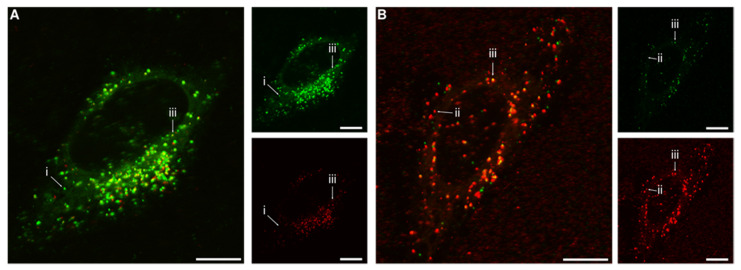
Subcellular distribution of the 40 nm and 100 nm particles. Cells were exposed for 24 h to both the 40 nm and 100 nm particles simultaneously and then observed using confocal fluorescence microscopy. (**A**) Concentration of 100 and 40 nm particles 20 and 100 μg/mL, respectively (0.060 nM and 4.7 nM; conditions correspond to the highest 40 nm particle concentration in Figure 2 and Figure S3). (**B**) Concentration of 40 and 100 nm particles 6.25 and 80 μg/mL, respectively (0.30 and 0.24 nM; conditions correspond to the highest 100 nm particle concentration in Figure 4 and Figure S4). The larger images show overlaps of both fluorescence colours, while the smaller images show the individual colours. (Green) 40 nm particles; (red) 100 nm particles. Arrows show examples of (i) 40 nm particle(s) (green) in the absence of 100 nm particles; (ii) 100 nm particle(s) (red) in the absence of 40 nm particles; (iii) 40 and 100 nm particles in the same location. The results show that the two particles often end up in the same location, but not always. All scale bars correspond to 10 μm.

Finally, we had a glimpse at the subcellular distribution of the two particles using confocal fluorescence microscopy (Figure 5). Previous studies have shown that both the 40 nm [52,55,66] and 100 nm [52,53,55,66] particles to a large extent end up in the lysosomes, both in the HeLa cells used here [53,55,66], as well as in other cell types [52,53]. We thus expect the same outcome, but whether the two particles will end up in the same lysosome (or other organelle) is less clear. To best represent the above results, we investigated the subcellular distribution under conditions corresponding to the largest impediment of uptake of the 100 nm particles by competing 40 nm particles (Figure 5A) as well as the largest promotion of uptake of the 40 nm particles by competing 100 nm particles (Figure 5B). Under both conditions, we observed the 40 nm (green) and 100 nm (red) particles in the same location (iii in Figure 5). Nevertheless, we also found 40 nm particles (green) in the absence of 100 nm particles (i) and, vice versa, 100 nm particles (red) in the absence of 40 nm particles (ii).

## 4. Conclusions

In summary, we show that competing 40 nm carboxylated polystyrene nanoparticles impede the uptake of 100 nm particles of the same material when both are exposed to HeLa cells at the same time (Figure 2 and Figure S3). Conversely, competing 100 nm particles promote the uptake of 40 nm particles (Figure 4 and Figure S4). Both of these observations are consistent with previous observations of silica nanoparticles (lacking a biomolecular corona) exposed to the same cell type [48], although we had to increase the concentration of competing smaller particles to a higher (number) concentration ratio to observe the effect. An analysis of uptake at single-cell-level, furthermore, suggests that the effect occurs for all cells of the population (Figure 3 and Figure S5).

The mechanisms underlying these observations still have to be determined, but our results do constrain the likely possibilities. Thus, the impediment of uptake of the 100 nm particles by competing 40 nm particles seems unlikely to stem from a saturation of “cell membrane adsorption sites” or “cell internalisation portals”. Furthermore, the promotion of uptake of the 40 nm particles by competing 100 nm is unlikely to result from the 100 nm particles promoting endocytosis in general. In fact, also the promotion of a specific endocytic mechanism via which both particles enter seems improbable. We cannot, however, rule out that the competing 100 nm particles induce an internalisation mechanism through which the 40 nm particles enter, but not the 100 nm particles themselves, although this seems a rather delicate possibility.

While we used two particles of the same material but different size, we note that our results should not necessarily be interpreted as a size effect. For example, the composition of the biomolecular corona is known to depend on particle size [49,67] and thus size is not the only variable being varied. However, *if* it were to be a size effect, then these results have a wider applicability than perhaps immediately apparent. As already noted in previous literature [48], most nanoparticle samples will exhibit some degree of polydispersity. Thus, the cellular uptake of a polydisperse sample may have to be interpreted as the outcome of a combination of the smaller particles impeding the uptake of the larger while, simultaneously, the larger particles promote the uptake of the smaller—a highly complex picture.

Results such as those exemplified here have implications for drug delivery using nano-sized carriers. For instance, simultaneous administration of a second particle could be used to promote cell uptake and hence, conceivably, the ultimate therapeutic effect. Conversely, one should also be aware of the potential for impeded uptake and a consequent possible loss of therapeutic effect.

## Data Availability

Data is contained within the article and Supplementary Material.

## Supplementary Materials

The following are available online at https://www.mdpi.com/article/10.3390/pharmaceutics14010136/s1, Supplementary Methods, Table S1: Nanoparticle dispersion characterisation, Figure S1: Nanoparticle dispersion size distributions, Table S2: Number of proteins in medium to nanoparticle surface area, Figure S2: Competing 40 nm particles do not affect the uptake of 100 nm particles at lower 40 nm particle concentrations, Figure S3: Competing 40 nm particles impede the uptake of 100 nm particles (repeat experiments of that shown in Figure 2), Figure S4: Competing 100 nm particles promote the uptake of 40 nm particles (repeat experiments of that shown in Figure 4), Figure S5: Uptake of 40 and 100 nm particles at individual cell level.
