# Supplementary material for: Simultaneous Exposure of Different Nanoparticles Influences Cell Uptake
Source: Pharmaceutics. 2022 Jan 6;14(1):136. doi: 10.3390/pharmaceutics14010136 (PMC8779877; doi:10.3390/pharmaceutics14010136)
Supplement: Supplementary file 1 [file pharmaceutics-14-00136-s001.zip › pharmaceutics-1424882-supplementary.pdf]

pharmaceutics

Supplementary Materials: Simultaneous Exposure of Different Nanoparticles Influences Cell Uptake

Isa de Boer, Ceri J. Richards and Christoffer Åberg

Supplementary Methods

Nanoparticle dispersion characterisation

The dispersions were characterised by dynamic light scattering using a Malvern ZetaSizer Nano ZS (Malvern Instruments, Malvern, UK). Dispersions of the particles were prepared in PBS and DMEM supplemented with serum and measured at room temperature. Three measurements were recorded for each sample and the results were analysed using ZetaSizer Software version 7.13 (Malvern Instruments, Malvern, UK).

Table S1. Nanoparticle dispersion characterisation using dynamic light scattering. The particles were dispersed in phosphate buffered saline (PBS), to characterise the original particles, and also in medium supplemented with serum, to characterise the dispersions exposed to cells. Particle concentrations were chosen to correspond to the *highest* ones used for the rest of the work as the risk for agglomeration should be higher for higher concentrations. The results are presented in terms of the *z*-average hydrodynamic diameter from cumulant analysis and in terms of the polydispersity index from cumulant fitting; the numbers correspond to the mean ± the standard deviation over three replicate measurements. Cumulant analysis is based on an assumption of a single population, which is neither applicable when the particles are dispersed in medium supplemented with serum, where proteins and other biomolecules are present, nor for the mixture of particles. We consequently also show size distributions from CONTIN analysis in Figure S1. Overall, the results here and in Figure S1 show that the original particles (in PBS) are fairly monodisperse and that when the particles are dispersed in medium no major agglomeration takes place. Furthermore, while the two particles cannot be individually detected in the mixture, it is clear that there is no major agglomeration also in these mixed dispersions.

Particle(s)	Concentration (μg/mL)	Medium	Diameter (nm)	Polydispersity Index
40 nm	100	PBS	56 ± 1	0.06 ± 0.004
		Medium	113 ± 1	0.24 ± 0.02
100 nm	80	PBS	110 ± 3	0.004 ± 0.003
		Medium	171 ± 2	0.20 ± 0.01
40 nm and 100 nm	100 and 20	Medium	133 ± 3	0.22 ± 0.01

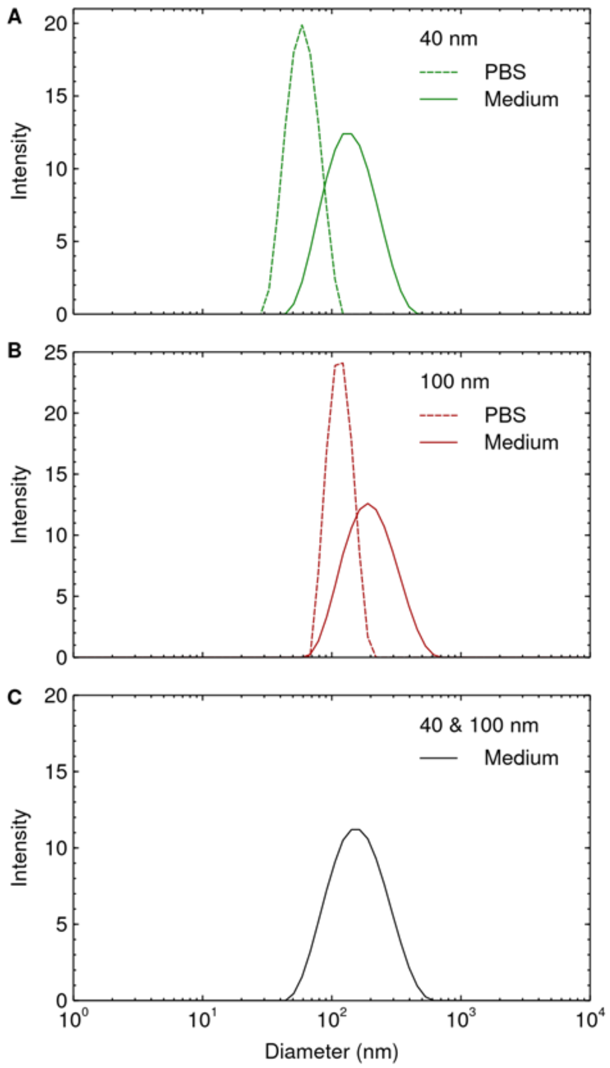

Figure S1. Nanoparticle size distributions by CONTIN analysis of dynamic light scattering data. Conditions correspond to those of Table S1. (**A**) 40 nm particles at a concentration of 100 µg/mL; (**B**) 100 nm particles at a concentration of 80 µg/mL; and (**C**) 40 nm particles and 100 nm particles at a concentration of 100 and 20 µg/mL, respectively. The original particles of both sizes are fairly monodisperse (PBS), while in medium supplemented with serum the distributions shift towards larger sizes. This is expected due to proteins and other biomolecules adsorbing to the particle surfaces to form a biomolecular corona, thereby increasing their size. However, one should not interpret the magnitude of the changes to the distributions in absolute terms, because of the complexity of the dispersion (*e.g.*, free biomolecules also contribute to the scattering). Other techniques can be used for an absolute quantification [1–3], but for our purposes it is sufficient to show a lack of agglomeration. For the mixture of the two particles, dynamic light scattering, as expected, does not allow deconvoluting the two contributions. Nevertheless we can conclude that there is no major agglomeration when the two particles are dispersed together.

Table S2. Sufficient number of proteins to cover the nanoparticle surface area. Our experiments were performed at 10% (bovine) serum concentrations, which corresponds to around 4 mg/mL. Given the complexity of serum it is impossible to give a complete calculation of the number of protein molecules in this dispersion. However, for our purposes the following estimate, based on a previously published estimate [4], will suffice: The main component of serum is serum albumin, which has a molecular weight of around 66.5 kDa. If all of the proteins were serum albumin, then 4 mg/mL would correspond to a molar concentration of around 60 μM. To estimate the molar concentration of adsorption sites on the particles we assume that a protein occupies a surface area of 5 nm × 5 nm = 25 nm^2 on the particle; this is less than the surface area reported to be occupied by human serum albumin and transferrin (see our previous estimate [4] and previous experimental literature [5–7] for details) and hence will lead to an *under*estimate of the (potential) excess of the number of proteins to surface area. Using this protein surface area, we find that the number of protein adsorption sites per particle is around 200 and 1300 for the 40 nm and 100 nm particles, respectively. Using a density of 1.05 g/mL for the polystyrene particles we can estimate the molar concentration of the particles and then the molar concentration of adsorption sites. Adding up the adsorption sites for the two particles thus yields the total number of adsorption sites (penultimate column). Note that the values reported in the table are rounded off, but we used the full precision of a computer for the calculation and so there may be minor discrepancies of intermediate results. The results show a clear excess of the number of proteins with regards to the total number of protein adsorption sites (final column). Note that we here consider the *highest* particle concentrations used in this work and thus there is an excess for all conditions.

**40 nm particles**			**100 nm particles**			**Total ads. sites molar conc. (μM)**	**Ratio molar conc. protein/ ads. sites**
**mass conc. (μg/mL)**	**molar conc. (nM)**	**molar conc. ads. sites (μM)**	**mass conc. (μg/mL)**	**molar conc. (nM)**	**molar conc. ads. sites (μM)**		
100	4.7	0.95	20	0.06	0.08	1.0	60
6.25	0.3	0.06	80	0.2	0.3	0.4	170

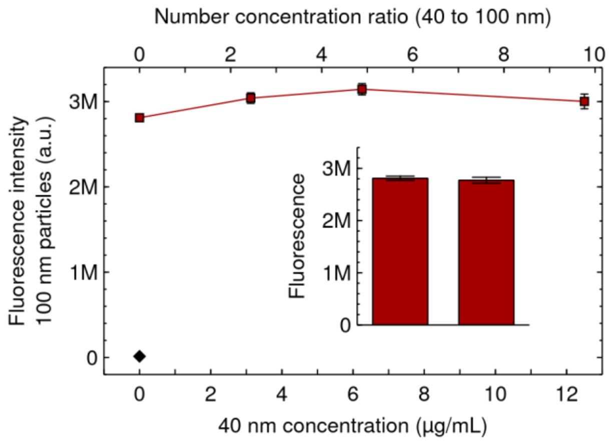

Figure S2. Competing 40 nm particles do not affect the uptake of the 100 nm particles at lower 40 nm particle concentrations. Cells were exposed for 24 h to both 100 nm and 40 nm particles simultaneously. The 100 nm particle concentration was kept constant at 20 µg/mL (0.060 nM), while the concentration of the 40 nm particles was varied (horizontal axes). (Main figure) Uptake of the 100 nm particles, showing that as the concentration of the competing 40 nm particles is increased, the uptake of the 100 nm particles remains roughly the same. (Inset) Cells exposed to the two dispersions of only the 100 nm particles (20 µg/mL; 0.060 nM) as a control for having achieved a similar concentration of the 100 nm particles (*cf.* Fig. 1A–B). Same y axis as the main figure and same data as shown in Figure 1B. Results are presented as the mean ± its standard error over 3 samples (some error bars are, however, smaller than the data symbols and are hence not visible). Diamond corresponds to control cells (not exposed to either of the particles). Overall, the results show that there is no effect on the uptake of 100 nm particles in the presence of competing 40 nm particles, at these lower concentrations of the competing 40 nm particles.

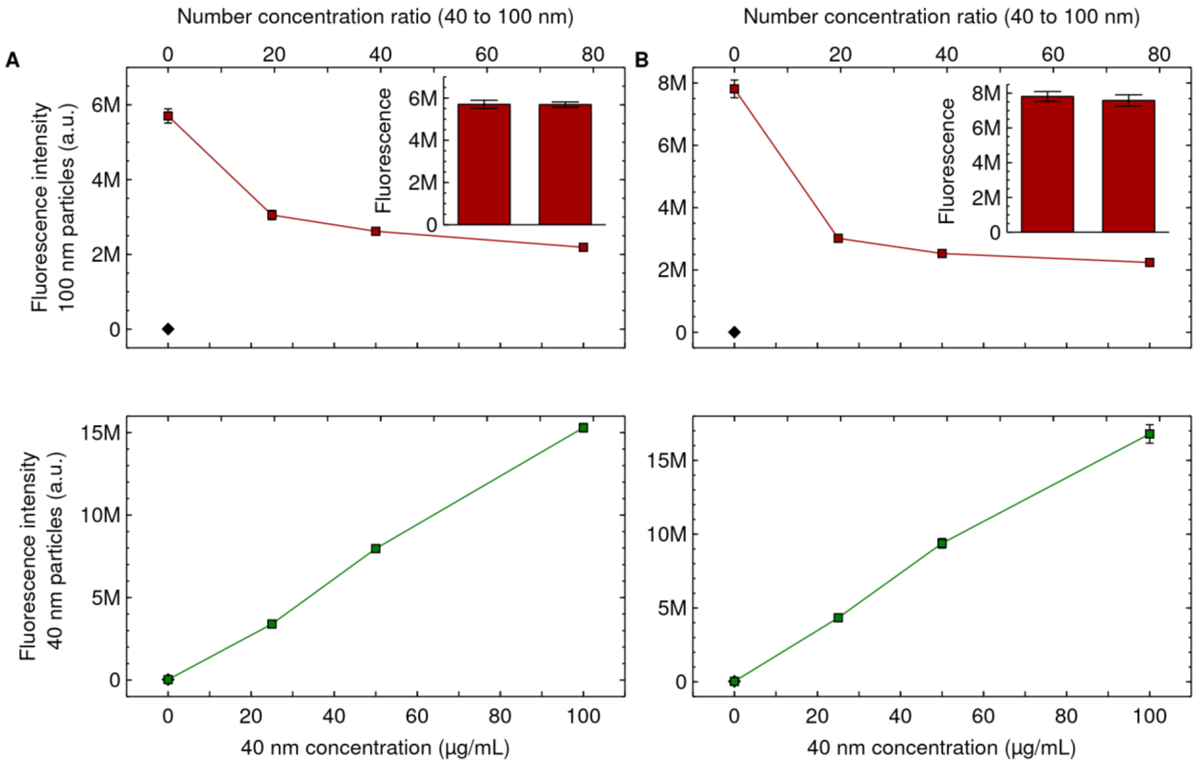

Figure S3. Competing 40 nm particles impede the uptake of 100 nm particles (repeat experiments of that shown in Figure 2). Cells were exposed for 24 h to both 100 nm and 40 nm particles simultaneously. The 100 nm particle concentration was kept constant at 20 µg/mL (0.060 nM), while the concentration of the 40 nm particles was varied (horizontal axes). (**A–B**) Results from independent experiments. (Upper graph) Uptake of the 100 nm particles, showing that as the concentration of the competing 40 nm particles is increased, the uptake of the 100 nm particles decreases. (Inset) Cells exposed to the two dispersions of only the 100 nm particles (20 µg/mL; 0.060 nM) as a control for having achieved a similar concentration of the 100 nm particles (*cf.* Fig. 1A–B). Same *y* axis as the main figure. (Lower graph) Uptake of the 40 nm particles, showing that the uptake of the competing 40 nm particles increases as their concentration is increased. Results are presented as the mean ± its standard error over 3 samples (most error bars are, however, smaller than the data symbols and are hence not visible). Diamond corresponds to control cells (not exposed to either of the particles). Overall, the results show that the uptake of 100 nm particles decreases in the presence of competing 40 nm particles.

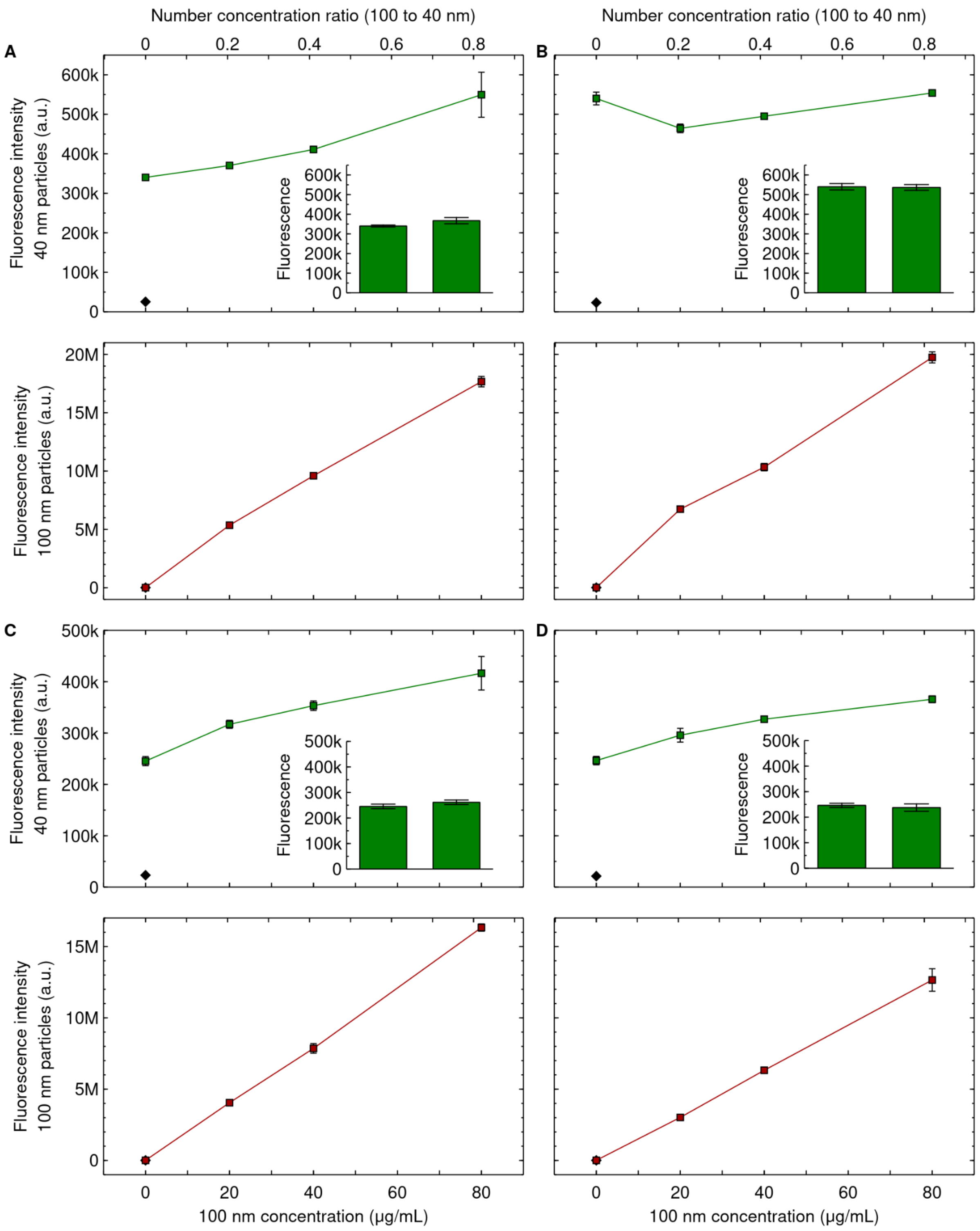

Figure S4. Competing 100 nm particles promote the uptake of 40 nm particles (repeat experiments of that shown in Figure 4). Cells were exposed for 24 h to both 40 nm and 100 nm particles simultaneously. The 40 nm particle concentration was kept constant at 6.25 μg/mL (0.30 nM), while the concentration of the 100 nm particles was varied (horizontal axes). (**A–D**) Results from independent experiments. (Upper graph) Uptake of the 40 nm particles, showing that as the concentration of the competing 100 nm particles is increased, the uptake of the 40 nm particles increases. (Inset) Cells exposed to the two dispersions of only the 40 nm particles (6.25 μg/mL; 0.30 nM) as a control for having achieved a similar concentration of the 40 nm particles (*cf.* Fig. 1A–B, but with the role of the 40 and 100 nm particles reversed). Same *y* axis as the main graph. (Lower graph) Uptake of the 100 nm particles, showing that the uptake of the competing 100 nm particles increases as their concentration is increased. Results are presented as the mean ± its standard error over 3 samples (most error bars are, however, smaller than the data symbols and are hence not visible). Diamond corresponds to control cells (not exposed to either of the particles). Overall, the results show that the uptake of 40 nm particles increases in the presence of competing 100 nm particles.

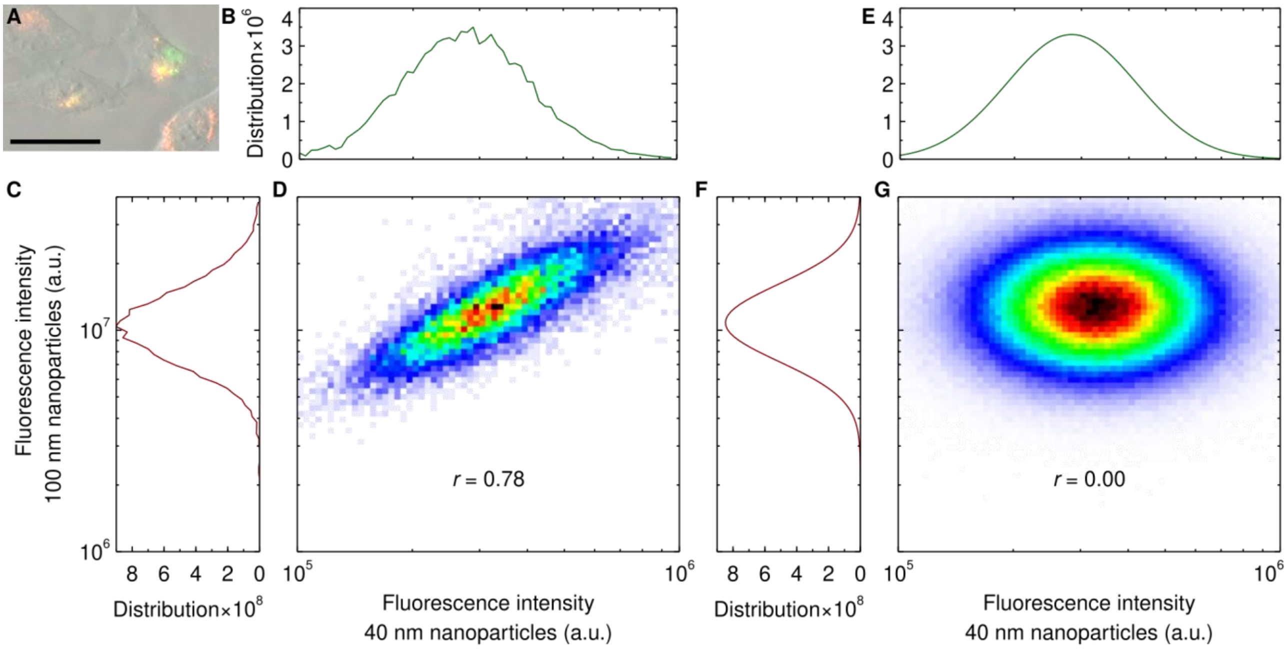

Figure S5. Uptake of 40 and 100 nm particles at individual cell level. (**A**–**D**) Experimental results. Cells were exposed for 24 h to both the 40 nm (6.25 µg/mL; 0.30 nM) and 100 nm (80 µg/mL; 0.24 nM) particles simultaneously (conditions correspond to the highest 100 nm particle concentration in Figure 4 and S4). (**A**) Microscopy of cells. (Grey) Phase-gradient contrast microscopy image showing the contour of the cells. (Green) 40 nm and (red) 100 nm particles. (Yellow) Overlap of the two particles. Note that the fluorescence intensity of the two particles is different, so the results cannot be interpreted in absolute terms. Scale bar corresponds to 50 µm. The results show that cells take up both nanoparticles. (**B–D**) Fluorescence of cells measured using flow cytometry. Distribution of cell fluorescence corresponding to the (**B**) 40 nm and (**C**) 100 nm particles. (**D**) Two-dimensional distribution of the two fluorescences. The results show a strong correlation between a cell having taken up one of the nanoparticles with it having taken up the other nanoparticle. (**E–G**) Theoretical distributions derived from fits to the experimental data (panels B–D). (**E–F**) Log normal distribution fits to the distribution of individual fluorescences (panels B–C). (**G**) Two-dimensional distribution derived under the assumption that two fluorescences are independent. To evaluate the distribution, 10^6 random samples were drawn from each of two individual distributions (panels E–F). The simulated results do not show any correlation between a cell having taken up one of the nanoparticles with it having taken up the other nanoparticle, in stark contrast to the experimental results (panel D). We can therefore conclude that experimentally the uptake of one particle is not statistically independent of the uptake of the other. Panels use the same scale where possible. All distributions are normalised such that their integral is 1. *r* denotes Pearson's correlation coefficient, which was evaluated in the interval shown to not bias the result from outliers. Overall, the results show that the uptake of one of the nanoparticles is strongly correlated with uptake of the other nanoparticle, at individual cell level.

References

1. WalczykD.; BombelliF.B.; MonopoliM.P.; LynchI.; DawsonK.A. What the Cell "Sees" in Bionanoscience. *J. Am. Chem. Soc.* **2010**, *132*, 5761–5768, doi:10.1021/ja910675v.
2. MonopoliM.P.; WalczykD.; CampbellA.; EliaG.; LynchI.; BombelliF.B.; DawsonK.A. Physical−Chemical Aspects of Protein Corona: Relevance to in Vitro and in Vivo Biological Impacts of Nanoparticles. *J. Am. Chem. Soc.* **2011**, *133*, 2525–2534, doi:10.1021/ja107583h.
3. KellyP.; ÅbergC.; PoloE.; O'ConnellA.; CookmanJ.; FallonJ.; Krpetić Željka; DawsonK.A. Mapping protein binding sites on the biomolecular corona of nanoparticles. *Nat. Nanotechnol.* **2015**, *10*, 472–479, doi:10.1038/nnano.2015.47.
4. VtyurinaN.; ÅbergC.; SalvatiA. Imaging of nanoparticle uptake and kinetics of intracellular trafficking in individual cells. *Nanoscale* **2021**, *13*, 10436–10446, doi:10.1039/d1nr00901j.
5. RöckerC.; PötzlM.; ZhangF.; ParakW.J.; NienhausG.U. A quantitative fluorescence study of protein monolayer formation on colloidal nanoparticles. *Nat. Nanotechnol.* **2009**, *4*, 577–580, doi:10.1038/nnano.2009.195.
6. JiangX.; WeiseS.; HafnerM.; RöckerC.; ZhangF.; ParakW.J.; NienhausG.U. Quantitative analysis of the protein corona on FePt nanoparticles formed by transferrin binding. *J. R. Soc. Interface* **2009**, *7*, S5–S13, doi:10.1098/rsif.2009.0272.focus.
7. MilaniS.; BombelliF.B.; PitekA.S.; DawsonK.A.; RädlerJ. Reversible versus Irreversible Binding of Transferrin to Polystyrene Nanoparticles: Soft and Hard Corona. *ACS Nano* **2012**, *6*, 2532–2541, doi:10.1021/nn204951s.
